# Dietary alterations modulate the microRNA 29/30 and IGF-1/AKT signaling axis in breast Cancer liver metastasis

**DOI:** 10.1186/s12986-020-00437-z

**Published:** 2020-03-23

**Authors:** Anuradha A. Shastri, Anthony Saleh, Jason E. Savage, Tiziana DeAngelis, Kevin Camphausen, Nicole L. Simone

**Affiliations:** 1 Department of Radiation Oncology, Sidney Kimmel Cancer Center at Thomas Jefferson University, Philadelphia, PA USA; 2 Center for Cancer Research, National Cancer Institute, Bethesda, MD USA

**Keywords:** Liver, Diet, Calorie restriction, Fasting, microRNA, Insulin signaling, Breast cancer metastases

## Abstract

**Background:**

Metastatic cancer is incurable and understanding the molecular underpinnings is crucial to improving survival for our patients. The IGF-1/Akt signaling pathway is often impaired in cancer leading to its progression and metastases. Diet modification is known to alter the IGF-1/Akt pathway and affect the expression of microRNA involved in tumor initiation, growth and metastases. Liver metastases are one of the most common type of metastases in breast and colon cancer. In the present study, we looked at the effect of diet modification on the expression of microRNA in normal liver and liver with breast cancer metastases using in vivo model.

**Methodology:**

6-month-old C57BL/6 J mice were put on either an ad libitum (AL) diet, or 40% calorie restricted (CR) diet or were fasted for 24 h (FA) before sacrifice. MicroRNA array analysis, western blot and qRT-PCR were performed using liver tissue to compare the treatment groups. A breast cancer model was also used to study the changes in microRNA expression in liver of a group of BALB/c mice orthotopically injected with 4 T1 cells in the mammary fat pad, put on either an AL or 30% CR diet. Liver and primary tumor tissues were used to perform qRT-PCR to compare the treatment groups.

**Results:**

MicroRNA array analysis showed significant changes in miRNA expression in both CR and FA conditions in normal liver. Expression of miR-29 and miR-30 family members was increased in both CR and FA. Western blot analysis of the normal liver tissue showed that CR and FA downregulated the IGF-1/Akt pathway and qRT-PCR showed that the expression of miR-29b, miR-29c, miR-30a and miR-30b were increased with CR and FA. Liver tissue collected from mice in the breast cancer model showed an increase in expression of miR-29b, miR-29c and miR-30b while tumor tissue showed increased expression of miR-29c, miR-30a and miR-30b.

**Discussion:**

Members of the miR-29 family are known to target and suppress IGF-1, while members of the miR-30 family are known to target and suppress both IGF-1 and IGF-1R. In the present study, we observe that calorie restriction increased the expression of miR-29 and miR-30 in both the normal liver as well as the liver with breast cancer metastases. These findings suggest that dietary alterations may play a role in the treatment of liver metastasis, which should be evaluated further.

## Introduction

Metastatic cancer is associated with disease that has spread beyond its initial site requiring constant treatment to prevent disease progression. Patients will ultimately succumb to metastatic breast cancer from either consequences of the disease or toxicity of treatment including cardiac toxicities and secondary malignancies. Liver metastases are among the most common types of metastases, representing more than 50% of breast and colorectal metastases, and are inescapably fatal [1, 2]. Few treatments exist to restrain these metastases, emphasizing the importance of identifying the novel molecular mechanisms driving them. The liver is a critical nutrient sensor and is rich in metabolic processes affected by dietary alterations evaluated in prior works [3–5], but has not yet been examined in the context of metastatic cancer. Caloric restriction (CR) has previously been shown to play a role in decreasing both primary tumor growth and metastases [6], and short-term or intermittent fasting have been shown to reproduce many of the physiologic changes observed during CR, such as slowing tumor growth and incidence [7–9]. It therefore follows that dietary alterations may play a role in managing liver metastases.

At the mechanistic level, microRNAs (miRNA), or small non-coding RNAs, play a role in cancer initiation, progression and metastases [10–14] and are altered by dietary interventions like fasting and caloric restriction [15–18]. In the present study, we used in vivo experiments to determine if dietary alterations affect miRNA expression in the normal liver or in liver metastases from breast cancer to identify potential novel targets for breast cancer liver metastases. We report global microRNA expression changes in liver tissue from normal mice fed ad libitum (AL), 40% CR diet, or following a 24-h fast. Here we show increased expression of miR-29 and miR-30 family members due to these dietary interventions in the normal liver and breast cancer liver metastases models. Multiple members of each of the miRNA family directly target and repress the IGF-1/IGF-1R signaling axis, which is associated with decreased cancer progression and metastases. Our findings support the use of dietary intervention in treatment and provide a novel targeting strategy for therapies in the future.

## Materials and methods

### Mice and dietary regimen

For the normal mice subjected to ad libitum (AL) feeding or dietary alterations, 15 6-month old C57BL/6 J mice were obtained through the National Institute of Aging (NIA) Aged Rodent Program under two feeding conditions (Charles River) on an Institutional Animal Care and Use Committee (IACUC) approved mouse protocol. One group of 10 mice was fed AL diet, and the remaining 5 mice were fed a calorie restriction (CR) diet of 60% normal intake. CR was initiated a week after arrival with a 10% restriction, increased to 25% restriction the next week, and to 40% restriction the following week, where it was maintained throughout the life of the animal. All mice were singly housed throughout the experiment and were fed NIH-31 food fortified with an NIA supplement for CR mice. Age-matched AL mice were fasted for 24 h (FA) before sacrifice.

To assess the effect of diet in a murine breast cancer model, 12-week-old BALB/c mice were acquired from Charles River Laboratories under an IACUC approved protocol at Thomas Jefferson University. At 13 weeks, all mice received orthotopic injections of 50,000 4T1 luciferase-tagged cells (a gift from Patricia Steeg) into the #4 mammary fat pad. Mice treated with CR were stepped down to 90% of their baseline chow intake for 5 days, followed by 2 successive 10% decreases every 5 days until a 30% total reduction was achieved (LabDiet 5010).

### Total RNA purification, array analysis, and real-time PCR

AL, CR, and fasted mice were sacrificed; the liver tissues were dissected and immediately placed in RNAlater (Ambion). RNA was isolated from the liver tissue using Trizol (Invitrogen) method. RNA concentration and quality were determined using Nanodrop and quantitative real-time PCR (qRT-PCR) was performed (Applied Biosystems 7500 fast) using the miScript SYBR Green PCR kit and miScript Primer Assay for miR-29 and miR-30 (Qiagen). miRNA levels were normalized to snord68 as an internal control. miRNA microarray analysis was performed by LC Sciences (Houston, TX) on 5 μg of total RNA as previously described [19].

### Immunoblotting

Total protein was extracted from 50 mg of RNA-later preserved mouse liver tissue. Tissue was added to 1 mL of SDS lysis buffer (1% SDS, 50 mM Tris pH 8.0, 10 mM EDTA, Protease inhibiter (Roche), and Halt Phosphatase Inhibitor (Thermo Scientific) in a lysing matrix D tube (MP Biomedicals). Tissue was homogenized using manufacturer’s suggested settings for a FastPrep-24 machine (MP Biomedicals). Samples were sonicated using a probe sonicator four times for 5 s each on ice and heated at 70 °C for 30 min with mixing. Lysates were cleared by centrifugation at 14,000 x g for 10 min at 4 °C. Protein concentration was determined using the BCA Protein Assay (Thermo Scientific). 40 μg of total protein was subjected to SDS-PAGE on a 4–12% gradient Bis-Tris gel (Invitrogen). Protein was transferred to a 0.2-μm PVDF membrane using the XCell transfer system (Invitrogen). Western blots were performed using the following antibodies: IGF-1 (Santa Cruz Biotechnology), IGF-1Rß (Cell Signaling Technology), phospho-IGF-1R tyrosine 1165/tyrosine 1166 (Sigma), IRS1 (Cell Signaling Technology), pan AKT (Cell Signaling Technology), phospho-AKT serine 473(Cell Signaling Technology), GAPDH (Santa Cruz Biotechnology).

### Bioinformatics and statistics

miRNA arrays were performed on 3 samples for each condition. Significance between normalized expressions from array samples was determined using Student’s *t*-test and *p* < 0.02. Data from the microarray experiments were collected and analyzed in accordance with the MIAME guidelines. MicroRNA expression profiles were analyzed using IPA software 9 core analysis function and microRNA target filter. miRNA target prediction was performed using Targetscan 5.1 and PICTAR. Unless otherwise stated, data represent the mean of 3 experiments, and error bars represent standard error of mean.

## Results

### Array analysis of miRNA changes during caloric restriction and fasting in mice

**Fig. 1:**
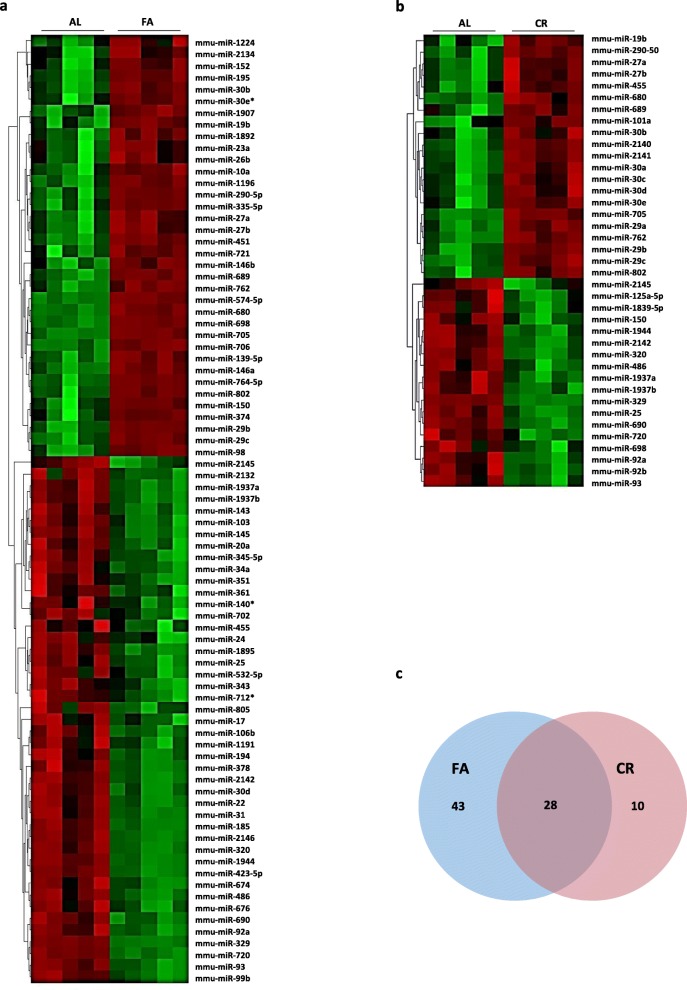
Diet modification changes the expressions of multiple microRNA in normal liver tissue of mice. MicroRNA expression patterns were detected by microarray from total RNA purified from normal liver tissue of C57BL/6 J mice following Ad. Libitum (AL) diet, 40% Caloric restriction (CR) diet or a 24-h fast (FA). **a** Heat map clustering of significantly changed microRNAs in normal livers of mice on AL or FA diet. **b** Heat map clustering of significantly changed microRNAs in normal livers of mice on AL or CR diet. **c** Venn diagram showing liver microRNA expression overlap between CR and FA diet groups

We detected global changes in miRNA expression using liver tissue from age-matched C57BL/6 J mice fed ad libitum (AL), 40% calorically restricted (CR), or 24 h fasted (FA) mice analyzed by miRNA arrays. These arrays show significant alterations in miRNA expression (Fig. 1a-b) as well as a 73% overlap between CR and FA in the miRNAs changed (Fig. 1c). Multiple microRNAs belonging to the highly conserved miR-29 and miR-30 families showed significantly increased expression after exposure to both dietary interventions.

In order to analyze the effect of miRNAs with altered expression in both conditions we used Ingenuity Pathway analysis (IPA) software’s miRNA target filter to identify experimentally observed targets reported in the literature. This list was filtered for miRNAs with detectable expression in mouse liver tissue in published arrays [20, 21]. Core IPA analysis of the target list revealed that one of the top networks altered involved cell growth and proliferation centered on IGF-1, EGF, and HGF growth factor signaling (Supplementary Figure 1). This is significant since many of the cancer prevention effects of CR are thought to involve repression of the pro-proliferation anti-apoptotic IGF-1/AKT/mTOR signaling axis [22, 23].

### CR increased expression of miR-29 and miR-30 family members and altered IGF-1 signaling in normal liver

The microRNA prediction algorithm TargetScan v6.0 and IPA’s miRNA target filter were utilized to identify predicted targets of the 26 microRNAs altered in both fasting and CR, yielding > 58,000 predicted targets. To focus this search, we filtered this gene list for targets of miR-29 and miR-30 families that are known to be involved in IGF-1 signaling.

**Fig. 2:**
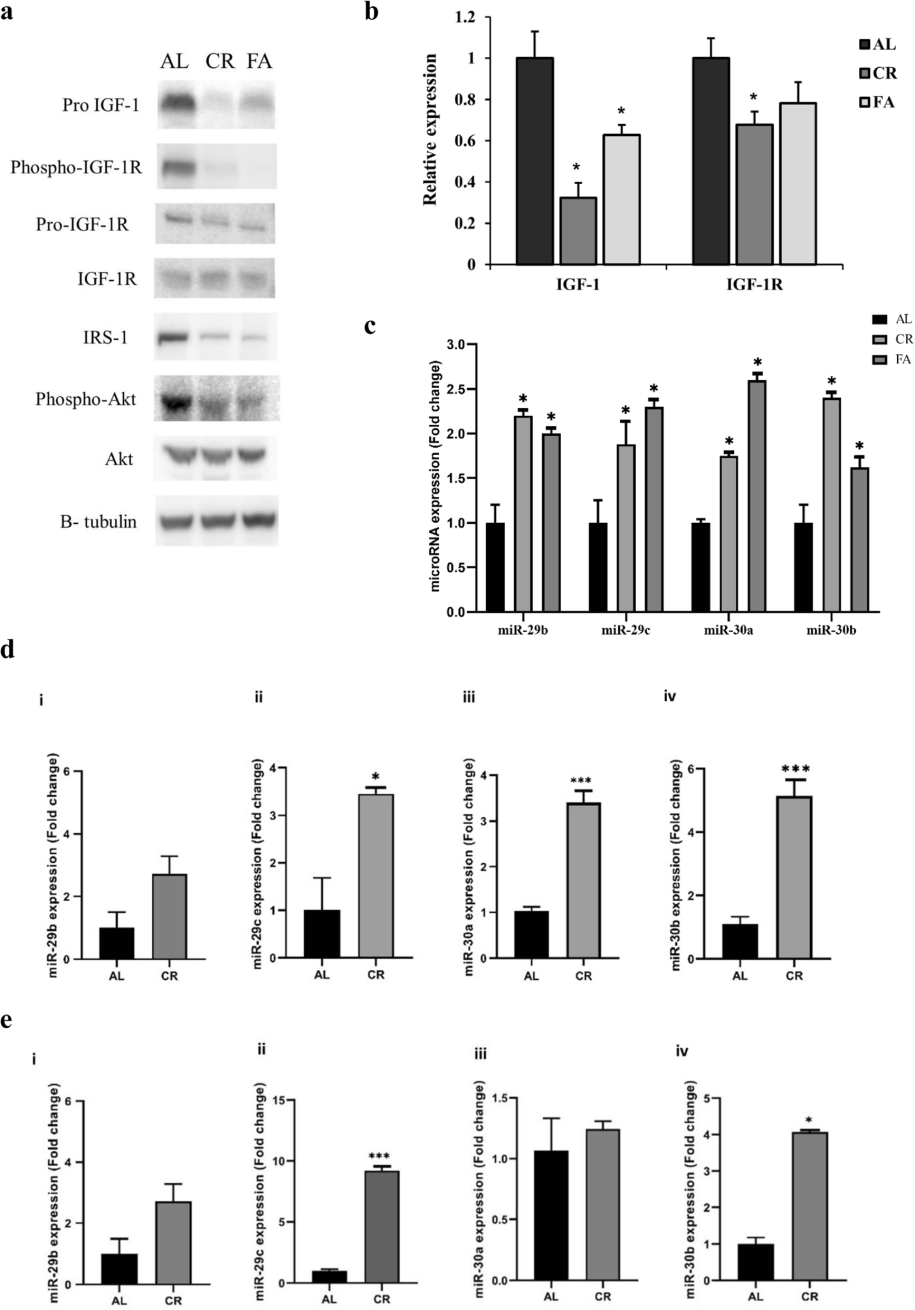
Diet modification using CR or FA repressed the IGF-1 pathway and altered the expression of miR-29 and miR-30. **a** Western blot analysis of IGF-1 pathway proteins from total protein purified from normal liver tissue of C57BL/6 J mice on AL, CR or FA diet. **b** qRT-PCR for expression of IGF-1 and IGF-1R in AL, CR, and FA normal liver tissue, mean ± SD (*- *p* < 0.05, *n* = 4). **c** Real time qPCR validation of miR-29b, miR-29c, miR-30a and miR-30b expression in normal liver of mice on AL, CR or FA diets, mean ± SD (*- *p* < 0.05, n = 4). **d** Expression of miR-29b (i), miR-29c (ii), miR-30a (iii) and miR-30b (iv) in primary breast cancer tumor of BALB/c mice on AL or CR (30%) diet represented by mean ± SE (***-*p* < 0.001 and *-*p* < 0.05, *n* = 3). **e** Expression of miR-29b (i), miR-29c (ii), miR-30a (iii) and miR-30b (iv) in liver tissue with metastases of breast cancer in BALB/c mice on AL or CR diets, represented by mean ± SE (***-*p* < 0.001 and *-*p* < 0.05, *n* = 3)

miR-29 represses p85 (PIK3R1) [24] and miR-30 targets JUN [25], both of which are important in cancer progression. We confirmed that multiple members of the IGF-1 signaling cascade, including pro-IGF-1, IGF-1R, IRS1, and phospho-Akt, are reduced in CR liver tissue (Fig. 2a). In CR and fasted tissue, IGF-1 and IGF-1R were reduced inversely to miR29 and miR-30 expression as validated by qRT-PCR (Fig. 2a, b and c).

### CR increases miR-29 and miR-30 expression in primary breast tumor and liver tissue with metastases

We assessed miR-29 and miR-30 expression in the primary breast tumor and liver tissue with breast cancer metastases of BALB/c mice fed AL or 30% reduced CR diets, to determine if CR effects the expression of these miRNAs. qRT-PCR was performed to survey miR-29 and miR-30 family member expression.CR increased miR-29b in the liver tissue by 2.5-fold compared to AL (Fig. 2e(i)) with no change in expression in the tumor (Fig. 2d(i)). CR also increased the expression of miR-29c in the tumor and liver tissue by ~ 3.4-fold and ~ 9-fold respectively (Fig. 2d (ii) and Fig. 2e (ii)). CR caused a 3.5-fold increase in the expression of miR-30a in tumor (Fig. 2d (iii)) and a 5-fold and 4-fold increase in miR-30b expression in tumor and liver tissue respectively (Fig. 2d (iv) and 2E (iv)).

## Discussion

Our initial hypothesis that microRNAs play a functional role in mediating the physiological effects of dietary restriction is supported by our observation of the changes in the unique global expression patterns of liver microRNAs of calorically restricted (− 40%) or 24-h fasted C57BL/6 J mice compared to AL controls. It must also be noted that fasting produces > 2 fold more alterations to microRNA expression than CR (71 vs. 38). We predict that the larger group of miRNAs altered by fasting is due to an acute response, while expression changes detected in the CR group represent stable changes due to longer-term dietary changes. However, 73% of the miRNA expression changes that were detected in the CR group were also altered in the fasted group, suggesting that they may be mechanistically important in pathways influenced by dietary alterations.

Systems analysis of experimentally reported targets of the group of miRNAs that were changed in both conditions using IPA software showed an enrichment in transcripts that, if repressed, would suppress several growth factor signaling pathways. This observation is in agreement with what is known in general about the cellular response to dietary restriction, whereby growth and reproduction is repressed in favor of maintenance and repair [26]. Expression of several family members of the miR-29 and miR-30 family was increased in both the fasted and CR tissues; and are associated with tumor suppressive processes. Specifically, the miR-29 family plays an inhibitory role in oncogenic metabolism affecting proliferation, angiogenesis, metastasis, and apoptosis. miR-29 has been shown to be tumor suppressive in more than 95% publications based on cancer studies [27]. Overexpression of miR-29a directly suppressed IGF-1R in the HepG2 Hepatocellular Carcinoma cell-line [28]. Park et al. in an in vitro study showed that members of the miR-29 family repressed p85α (PI3K) and upregulated p53 [24]. miR-30 family members act as both tumor suppressive and oncogenic miRNAs depending on the type of cancer. As a tumor suppressor in breast cancer, gastric cancer, pancreatic cancer and renal cell carcinoma, miR-30 prevents proliferation, invasion and metastasis [29–32] by targeting IGF-1R [31].

Many primary tumors spread to the liver, with greater than 50% of colorectal tumors metastasizing to the liver [2]. Other more common cancers that produce liver metastases include breast cancer, lung cancer and pancreatic cancer among others [32]. Since the members of the miR-29 and miR-30 family repress the IGF-1 signaling pathway, we explored the capacity of diet to alter metastatic tumor biology. CR increased the expression of miR-29b, miR-30a and miR-30b in the tumor, and increased expression of miR-29b, miR-29c and miR-30b in the liver tissue with metastases from breast tumor. We believe that alterations to miR-30a expression in primary breast cancers not observed in the liver are due to differences in the two environments that drive different expression pathways leading to miR-30 family expression. Here we show that CR leads to increased expression of miR-29 and miR-30 in the both the normal liver, primary breast tumor and liver with metastases from BC. Both these microRNAs repress IGF-1, while IGF-1R was a target of miR-30 alone. These findings are in agreement with our previous studies in an in vivo model of triple negative breast cancer (TNBC) that showed that at both the mRNA and protein level these genes are downregulated by CR alone or in combination with other treatments [6, 33]. Diet modification has also been shown to downregulate the IGF-1 signaling in other cancers including prostate, pancreatic and colon cancer thereby delaying their progression [34–36]. Many studies have demonstrated the role of IGF-1/ IGF-1R signaling pathway in invasion and metastasis [37–39].

The liver produces 90% of all IGF-1 and we observe that CR represses IGF-1 and IGF-1R via regulation by miR-29 and miR-30. Taken together we hypothesize that CR combined with other treatment may be able to prevent progression of liver metastasis via the upregulation of miR-29 and miR-30 and downregulation of the IGF-1/AKT/mTOR signaling pathway. Using a dietary intervention to downregulate the IGF-1 pathway could represent an ideal strategy to employ in clinical practice since the downregulation of this pathway is known to decrease cancer progression and metastases.

## Supplementary information

**Additional file 1.**

## Data Availability

All data generated or analyzed during this study are included in this published article (and its supplementary information files).

## Abbreviations

AL: Ad. Libitum
CR: Calorie restriction
EGF: Epidermal growth factor
HGF: Hepatocyte growth factor
IGF-1: Insulin-like growth factor-1
IGF-1R: Insulin-like growth factor-1 receptor
IPA: Ingenuity pathway analysis
miR, miRNA: microRNA
TNBC: Triple negative breast cancer
