# Supplementary figures and images for: Dietary alterations modulate the microRNA 29/30 and IGF-1/AKT signaling axis in breast Cancer liver metastasis
Source: Nutr Metab (Lond). 2020 Mar 23;17:23. doi: 10.1186/s12986-020-00437-z (PMC7092508; doi:10.1186/s12986-020-00437-z)

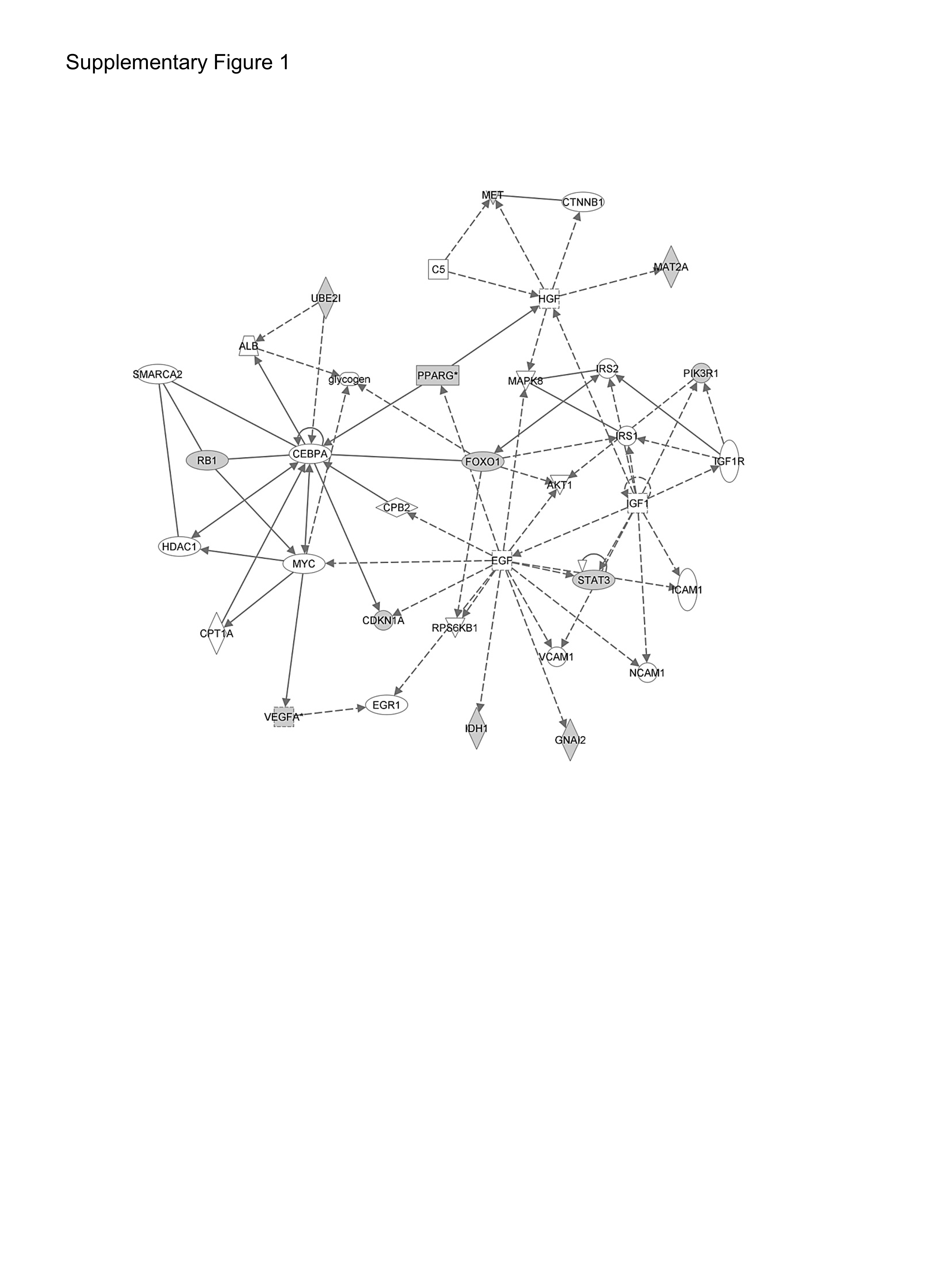

Supplement: Supplementary file 1 — Additional file 1. [file 12986_2020_437_MOESM1_ESM.tif]
